# Derivation of Transgene-Free Human Induced Pluripotent Stem Cells from Human Peripheral T Cells in Defined Culture Conditions

**DOI:** 10.1371/journal.pone.0097397

**Published:** 2014-05-13

**Authors:** Yoshikazu Kishino, Tomohisa Seki, Jun Fujita, Shinsuke Yuasa, Shugo Tohyama, Akira Kunitomi, Ryota Tabei, Kazuaki Nakajima, Marina Okada, Akinori Hirano, Hideaki Kanazawa, Keiichi Fukuda

**Affiliations:** 1 Department of Cardiology, Keio University School of Medicine, Shinjuku, Tokyo, Japan; 2 Japan Society for the Promotion of Science, Chiyodaku, Tokyo, Japan; 3 Department of Cardiovascular surgery, Keio University School of Medicine, Shinjuku, Tokyo, Japan; University of Milan, Italy

## Abstract

Recently, induced pluripotent stem cells (iPSCs) were established as promising cell sources for revolutionary regenerative therapies. The initial culture system used for iPSC generation needed fetal calf serum in the culture medium and mouse embryonic fibroblast as a feeder layer, both of which could possibly transfer unknown exogenous antigens and pathogens into the iPSC population. Therefore, the development of culture systems designed to minimize such potential risks has become increasingly vital for future applications of iPSCs for clinical use. On another front, although donor cell types for generating iPSCs are wide-ranging, T cells have attracted attention as unique cell sources for iPSCs generation because T cell-derived iPSCs (TiPSCs) have a unique monoclonal T cell receptor genomic rearrangement that enables their differentiation into antigen-specific T cells, which can be applied to novel immunotherapies. In the present study, we generated transgene-free human TiPSCs using a combination of activated human T cells and Sendai virus under defined culture conditions. These TiPSCs expressed pluripotent markers by quantitative PCR and immunostaining, had a normal karyotype, and were capable of differentiating into cells from all three germ layers. This method of TiPSCs generation is more suitable for the therapeutic application of iPSC technology because it lowers the risks associated with the presence of undefined, animal-derived feeder cells and serum. Therefore this work will lead to establishment of safer iPSCs and extended clinical application.

## Introduction

Induced pluripotent stem cells (iPSCs) are expected to provide new cell sources for revolutionary therapies[1]. In initial studies of generating human iPSCs, human fibroblasts were reprogrammed using forced expression of reprogramming factors[2]. Further studies subsequently showed successful reprogramming of several types of human somatic cells[3]. A range of donor cell types has been used to generate iPSCs; however, blood cells have attracted much of the attention because of the ease of sampling. Of these, T cells are a unique cell source for the generation of iPSCs because T cell-derived iPSCs (TiPSCs) have monoclonal T cell receptor (TCR) gene rearrangements in their genomes[4]–[7] and they can be differentiated into antigen-specific T cells for use in novel immunotherapy[8], [9]. Furthermore, clones and descendants of TiPSCs can be identified via the unique rearrangement patterns in the TCR gene locus[5].

Human T cells have been successfully reprogrammed with the exogenous expression of four transcription factors, *OCT3/4*, *SOX2*, *KLF4*, and *c-MYC* [4]–[7], [10]. Among these methods, Sendai virus (SeV) is an RNA virus of the Paramyxoviridae family that replicates in the cytoplasm and does not go through a DNA phase that can integrate into the host genome[11]–[13]. iPSCs generation using a combination of activated T cells and SeV has therefore showed benefit due to the high induction efficiency and lack of genome insertion of exogenous genes[5].

On another front, the application of culture systems to reduce potential risks of unpredictable agent becomes increasingly vital for applying iPSCs clinically[14]. The initial culture system for ESC generation used fetal calf serum in the culture medium and mouse embryonic fibroblast (MEF) as a feeder layer, introducing possibilities for transferring exogenous antigens, unknown viruses, or zoonotic pathogens to the generate cell populations[15]. An animal product-free medium that supports the derivation and long-term feeder-independent culture of human ESCs was recently developed[16], and in the field of iPSC generation, many efforts have been made to remove potentially harmful agents from the culture system[17]–[20].

Therefore, we investigated generating transgene-free human TiPSCs by applying a combination of SeV and activated T cells into defined culture medium and feeder-free conditions towards future clinical application of TiPSCs.

## Materials and Methods

### Cell Culture and SeV Infection

Blood samples were obtained from healthy donors. Samples were obtained only after the donors had provided written informed consent for blood sampling for the subsequent generation of iPSC. The handling these samples was approved by the Ethics Committee of Keio University (20-92-5). In addition, the study conformed to the principles outlined in the Declaration of Helsinki regarding the use of human tissue or subjects.

Peripheral blood mononuclear cells (PBMCs) were obtained from healthy donors (four men [31, 30, 30, and 31 years of age] and one 30-year-old woman) by the centrifugation of heparinized blood over a Ficoll-Paque PREMIUM (GE Healthcare) gradient, according to the manufacturer’s instructions. PBMCs were cultured at 37°C in 5% CO_2_ with plate-bound anti-CD3 monoclonal antibody (BD Pharmingen) in GT-T502 medium (KOHJIN BIO) that contained rIL-2 at 175 JRU/ml. After 5 days of culture, activated PBMCs were collected and transferred at 1.5×10^6^ cells/well to a new 6-well plate coated with the anti-CD3 mAb, and incubated for an additional 24 hours. Then, cells were transduced with the CytoTune iPS Reprogramming Kit (DNAVEC) that contained SeV vectors individually encoding for each of *OCT3/4*, *SOX2*, *KLF4*, and *c-MYC* at an MOI of 10. After 24 hours of infection, the medium was changed to fresh GT-T502 medium. At 48 hours post-infection, the cells were collected and transferred to a 10 cm-dish coated with Matrigel (BD Biosciences) in mTeSR1 media (StemCell Technologies Inc.). After an additional 24 h incubation, the medium was changed to new mTeSR1 medium and was changed thereafter daily until the colonies were selected. The TiPSCs generated were also maintained on Matrigel in mTeSR1 medium. The mTeSR1 medium was changed every other day, and the cells were passaged using dissociation solution for human ES/iPS Cells (CTK solution, ReproCELL) every 6–7 days. M-TiPSCs1 and M-TiPSCs2 were derived from a 31 year old man. In this series of experiments, the human ESC line KhES-2 [21] was used as a positive control and was cultured using the same protocol as for the iPSC culture described above. The KhES-2 cells were obtained from the Department of Development and Differentiation, Institute for Frontier Medical Sciences (Kyoto University) and used in line with the Guidelines for Derivation and Utilization of Human Embryonic Stem Cells of the Ministry of Education, Culture, Sports, Science, and Technology, Japan.

### ALP and Immunofluorescence Staining

ALP staining was performed with the ALP substrate (1-step NBT/BCIP; Pierce) after fixation with 4% paraformaldehyde (PFA; MUTO Pure Chemicals, Japan). Immunofluorescence staining was performed using the following primary antibodies: anti-NANOG (RCAB0003P, ReproCELL), anti-OCT3/4 (sc-5279, Santa Cruz), anti-SSEA 3 (MAB4303, Millipore), anti-SSEA 4 (MAB4304, Millipore), anti-Tra-1–60 (MAB4360, Millipore) and anti-Tra-1–81 (MAB4381, Millipore), anti-human smooth muscle actin (IR61161, DAKO), anti-human Sox17 (AF1924, R&D Systems), or anti-Nestin (N5413, Sigma). The fluorescence signals were detect using a conventional fluorescence laser microscope (IX70; Olympus) equipped with a color charge-coupled device (CCD) camera (CS220; Olympus). DAPI (Molecular Probes) and Hoechst (33342; Lonza) were used for nuclear staining. The secondary antibodies used were: anti-rabbit IgG and anti-mouse IgG and IgM conjugated with Alexa Fluor 488 or Alexa Fluor 568 (Molecular Probes).

### Quantitative PCR Analysis

Total RNA samples were isolated using TRIZOL reagent (Invitrogen) and RNase-free DNase I (Qiagen), according to the manufacturer’s instructions. The concentration and purity of the RNA were determined using an ND-1000 spectrophotometer (Nanodrop), and the cDNA was synthesized using the Superscript First-Strand Synthesis System (Invitrogen). Quantitative PCR (QT-PCR) was performed using a 7500 real-time PCR system (Applied Biosystems), with SYBR Premix ExTaq (Takara, Otsu, Japan). The amount of mRNA was normalized against that of *GAPDH* mRNA. Primer sequences and cycling conditions are listed in Table S1.

### Bisulfite Sequencing

Genomic DNA was isolated from bulk cell culture samples or undifferentiated colonies. A total of 5 µg DNA was used as the input for bisulfite conversion with EZ DNA Methylation-Gold Kit (ZYMO RESEARCH) according to the manufacturer’s protocol. The converted DNA was used as a template for conventional nested PCR to amplify the regions of the *OCT3/4* and *NANOG* promoters[22]. The primers were specific for conversion of the sense DNA strands (Table.S1). The purified PCR products were TA-cloned into the pGEM-T vector (Promega), and insert sequences of randomly picked clones were analyzed using the ABI 3700 DNA analyzer (Applied Biosystems).

### Analysis of TCR Gene Rearrangement in Genomic DNA

Genomic DNA was extracted from approximately 5×10^6^ cells using a QIAamp DNA mini kit (QIAGEN) according to the manufacturer’s instructions. For *TCRB* gene rearrangement analysis, PCR was performed according to BIOMED-2 protocols[23]. The dominant band within the expected size range was purified using a Wizard SV Gel and PCR Clean-Up System (Promega) and the purified PCR products were TA-cloned into the pGEM-T vector (Promega). The insert sequences of randomly picked clones were analyzed using the ABI 3700 DNA analyzer (Applied Biosystems). V, D, and J segment usages were identified by comparison to the ImMunoGeneTics (IMGT) database (http://www.imgt.org/) and by using an online tool (IMGT/V-QUEST) [24]. Gene-segment nomenclature follows IMGT usage.

### Global Gene Expression Analysis

For transcriptional profiling, total RNA was isolated from TiPSCs using the RNeasy Mini Kit (Qiagen). Cyanine-labeled antisense RNA was amplified using the Quick Amp Labeling Kit (Agilent), hybridized with the Gene Expression Hybridization Kit onto a Whole Human Genome Oligo Microarray (Agilent), and analyzed using the Agilent Microarray Scanner. The data were analyzed with the GeneSpring GX12.0 software (Agilent). Two normalization procedures were applied. Initially, the signal intensities with values less than 1 were assigned a value of 1. Then, each chip was normalized to the 50th percentile of the measurements taken from that chip. Finally, each gene was normalized to the median of that gene in the respective controls, to enable comparisons of relative changes in gene expression levels between different conditions. The microarray data in this experiment have been deposited in GEO and given the series accession number GSE56234.

### Teratoma Formation

TiPSCs (at a concentration corresponding to 25% of the cells from a confluent 150-mm dish per mouse) were injected into the testis of SCID mice (CLEA, Japan). Prior to injection, mice were anesthetized using a mixture of ketamine (50 mg/kg), xylazine (10 mg/kg), and chlorpromazine (1.25 mg/kg). Adequate anesthesia was maintained by monitoring the heart rate, muscle relaxation, and loss of a sensory reflex response (i.e. no response to tail pinching) in mice. At around 8 weeks after injection, mice were killed by cervical dislocation and the teratomas were dissected, fixed in 10% PFA overnight, and embedded in paraffin. The sections were stained with hematoxylin and eosin. All experiments were performed in accordance with the Keio University Animal Care Guidelines and were approved by the Ethics Committee of Keio University (20-041-4), which conforms to the Guide for the Care and Use of Laboratory Animals published by the US National Institutes of Health (NIH Publication no. 85-23, revised 1996).

### In vitro Differentiation

Cells were harvested using CTK solution (ReproCELL), and transferred to ultralow attachment plates (Corning) in hiPSC medium without bFGF. After 8 days, aggregated cells were plated onto gelatin-coated tissue culture dishes and incubated for an additional 8 days. The cells were incubated at 37°C in 5% CO_2_ and the medium was replaced every other day.

### Chromosome Karyotyping

TiPSCs treated with colcemid solution (60 ng/ml) were cultured for 5 hours at 37°C to stop cell growth in metaphase. These cells were then incubated in 0.075 M KCl solution for 10 min at room temperature, followed by fixation in Carnoy’s solution. Fixed cells were sent to Nihon Gene Research Laboratories Inc. (Sendai, Japan) for analysis.

## Results

### Generation of Human TiPSCs under a Defined Culture Condition

**Figure 1:**
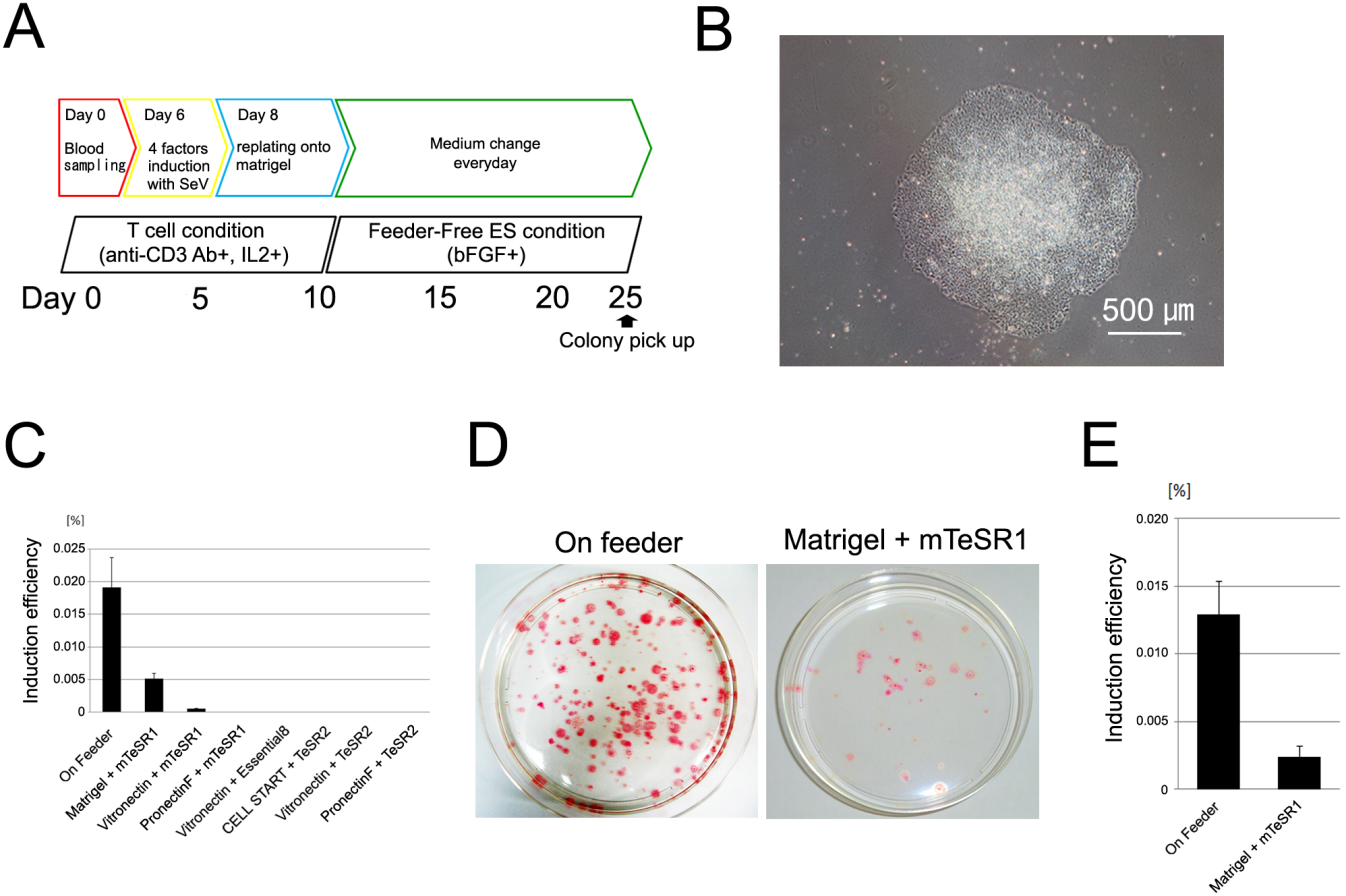
Generation of human TiPSCs under defined conditions. (A): Strategy used in the present study for reprogramming T cells. (B): Typical ESC-like TiPSC colony on day 25 after blood sampling under the defined culture condition. (C): Comparison of reprogramming efficiencies between the culture system using a feeder cell layer and that using defined culture conditions. Data show the mean ± s.d. (D): Comparison of representative 10-cm dishes stained for ALP (red spots) between feeder layer condition and defined culture condition (Matrigel) on day 25. (E): Comparison of reprogramming efficiencies between a culture system using a feeder cell layer and one using Matrigel and mTeSR1 medium for samples from five donors. Data show the mean ± s.d.

First, we attempted to find a culture system involving alternatives to feeder cells in the generation of human TiPSCs. Previously, Matrigel[25], pronectin F[26], vitronectin[18], and CellStart[27] have been used as feeder cell substitutes for the successful culture of human pluripotent cells. Therefore we examined the feasibility of generating TiPSCs on these substitutes in a chemically defined culture medium, mTeSR1 or TeSR2. Activated human peripheral blood mononuclear cells (PBMCs) were infected with SeV vectors expressing *OCT3/4*, *SOX2*, *KLF4*, and *c-MYC*, and then replated onto feeder cells or one of the substitutes (Fig. 1A). Within 3 weeks of infection, we identified colonies that resembled human ESCs using mTeSR1 and Matrigel (Fig. 1B) or vitronectin. Although the efficiency was generally lower than culturing on feeder cell layers, the Matrigel and mTeSR1 combination resulted in higher efficiency than the other feeder-free conditions (Fig. 1C,1D), with successful iPSC generation from all donors (Fig. 1E). After picking these colonies on Day 25 from blood sampling, we established cell lines that maintained human ESC-like morphology.

### Characterization of TiPSCs Generated under a Defined Culture Condition

**Figure 2:**
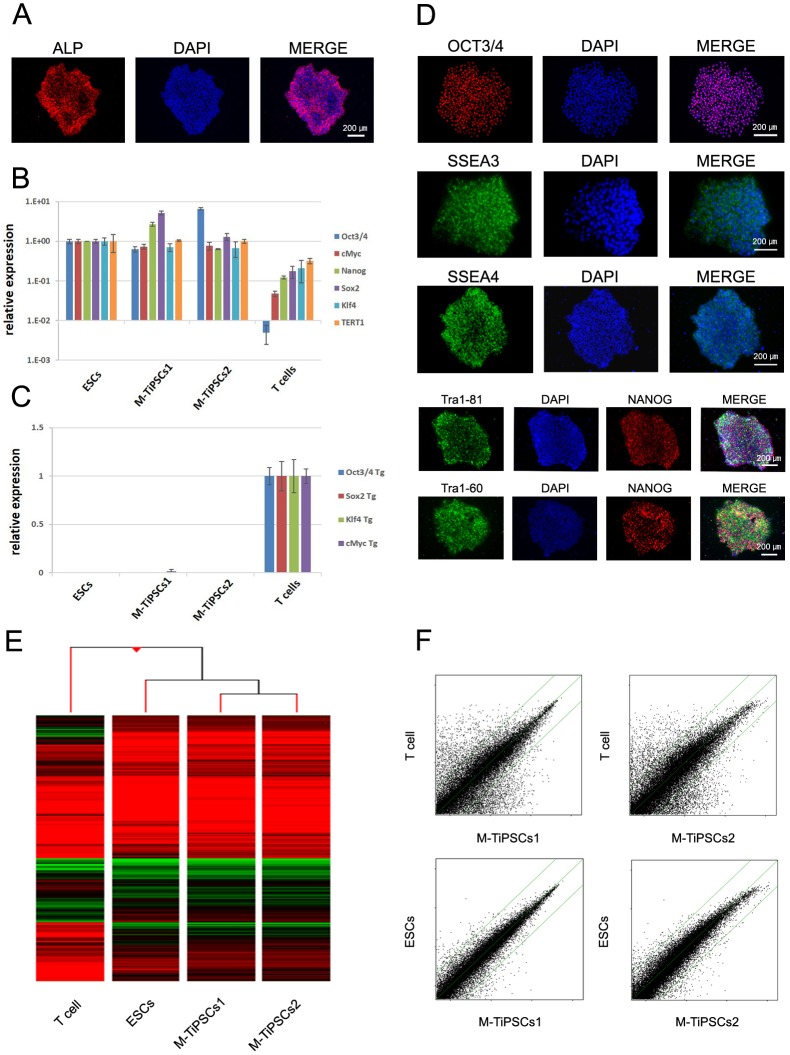
Characterization of M-TiPSCs generated under a defined culture condition. (A): ALP staining in M-TiPSCs. (B): QT-PCR analyses of M-TiPSCs for the ESC marker genes *OCT3/4*, *NANOG*, *SOX2*, *KLF4*, *c-MYC*, and *TERT1*. (C): QT-PCR analyses of M-TiPSCs for the transgenes, *OCT3/4*, *SOX2*, *KLF4*, and *c-MYC*. (D): Immunofluorescence staining for pluripotency and surface markers (NANOG, OCT3/4, SSEA3, SSEA4, TRA-1–60, and TRA-1–81) in M-TiPSCs1. (E): Heat map analyses of M-TiPSCs, ESCs, and the parental human T cells. (F): Scatter plots comparing the global gene expression profiles of M-TiPSCs with those of T cells and ESCs.

**Figure 3:**
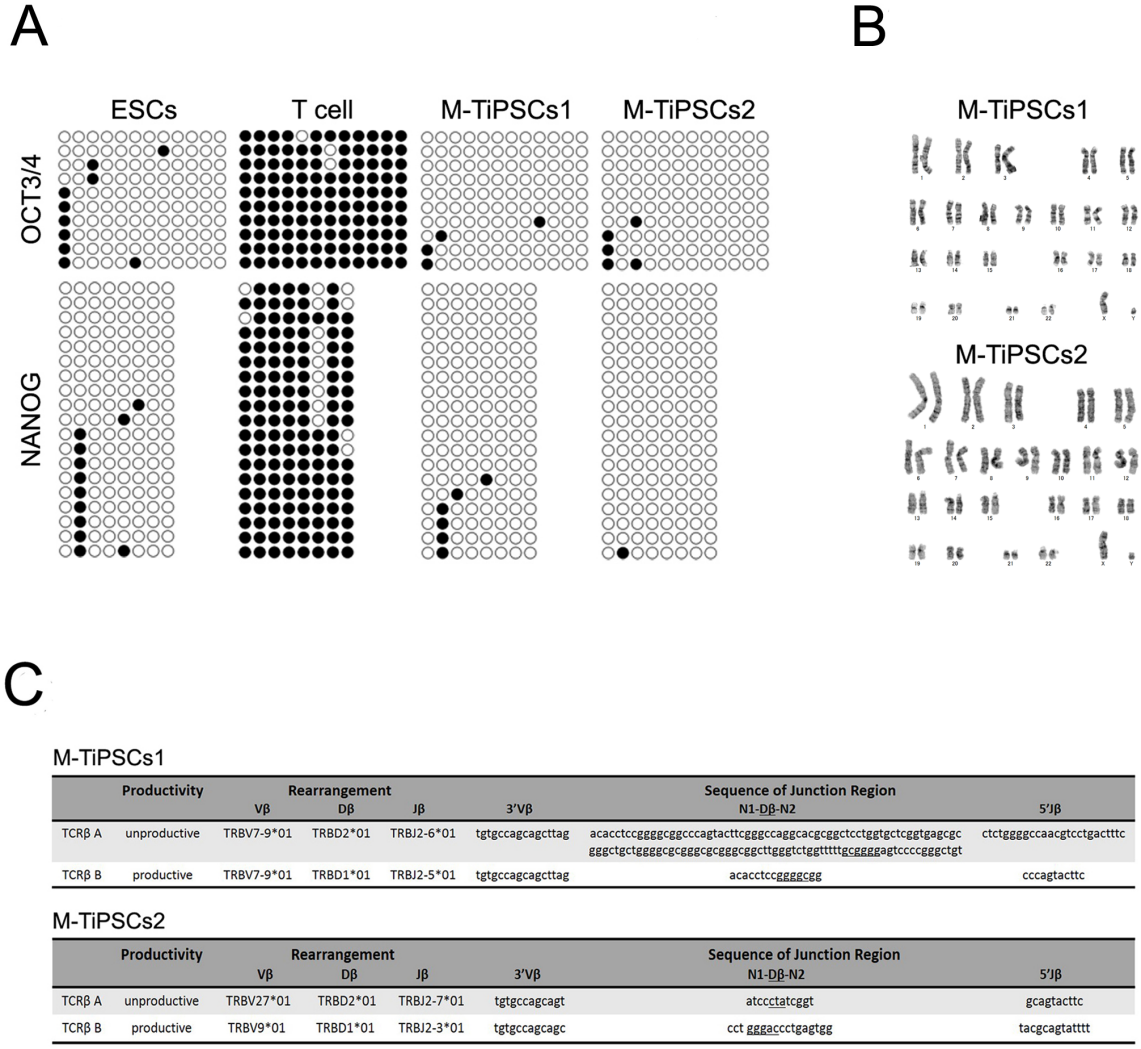
Analysis of TiPSCs genome modification and karyotype. (A): Bisulfite sequencing analysis of the *NANOG* and *OCT3/4* promoter regions in peripheral T cells, ESCs, and M-TiPSCs. Each row of circles for a given amplicon represents the methylation status of the CpG dinucleotides in one bacterial clone for that region. Open circles represent unmethylated CpGs and closed circles represent methylated CpGs. (B): G-band analysis for karyotypes of M-TiPSCs generated under a defined culture condition. M-TiPSCs1 and M-TiPSCs2 at passages 6 and 15, respectively, were used for G-band analysis. (C): Analysis of TCR rearrangements. V, D, and J segment usages in the *TCRB* gene locus were sequenced and identified by comparison to the international ImMunoGeneTics information system database. M-TiPSCs showed rearrangements of Vβ/Dβ1,2 and Dβ1,2/Jβ2.

To confirm that these TiPSCs generated on Matrigel (M-TiPSCs) had the characteristics of typical ESCs and iPSCs, we analyzed stem cell marker expression at the protein and mRNA levels, by DNA chips, and with bisulfite sequencing of the *NANOG* and *OCT3/4* promoters. Immunostaining and QT-PCR analyses revealed typical pluripotent marker expression in the M-TiPSCs (Fig. 2A,2B,2D, Fig.S1A). On the other hand, QT-PCR analysis showed no SeV transgene expression in the M-TiPSCs (Fig. 2C). Heat map and scatter plot analyses of human peripheral circulating T cells, ESCs, and M-TiPSCs showed that the global gene expression profiles of M-TiPSCs were overall similar to ESCs, and different from the parental human T cells (Fig. 2E, 2F). Bisulfite sequencing of M-TiPSCs showed that CpGs in the promoter regions of *NANOG* and *OCT3/4* were predominantly unmethylated in the M-TiPSCs, as is the case in ESCs (Fig. 3A). Although previous reports have raised the possibility of leading chromosomal instabilities of human ESCs in feeder-free cultures [28], [29], G-band analysis in this study revealed that the M-TiPSCs had normal karyotypes (Fig. 3B). These results suggested that SeV-mediated gene transfer on our defined culture conditions successfully reprogrammed human T cells into iPSCs that are similar to ESCs.

### Analysis of TCR Rearrangements

To confirm that the M-TiPSCs were derived from mature T cells, we analyzed the TCR rearrangements by sequencing the V, D, and J segment usages in the T-cell receptor beta (*TCRB*) gene locus. The specific rearrangement pattern identified in the *TCRB* gene locus of the M-TiPSCs (Fig. 3C) confirmed their mature T cell origins.

### In vitro and In vivo Differentiation

**Figure 4:**
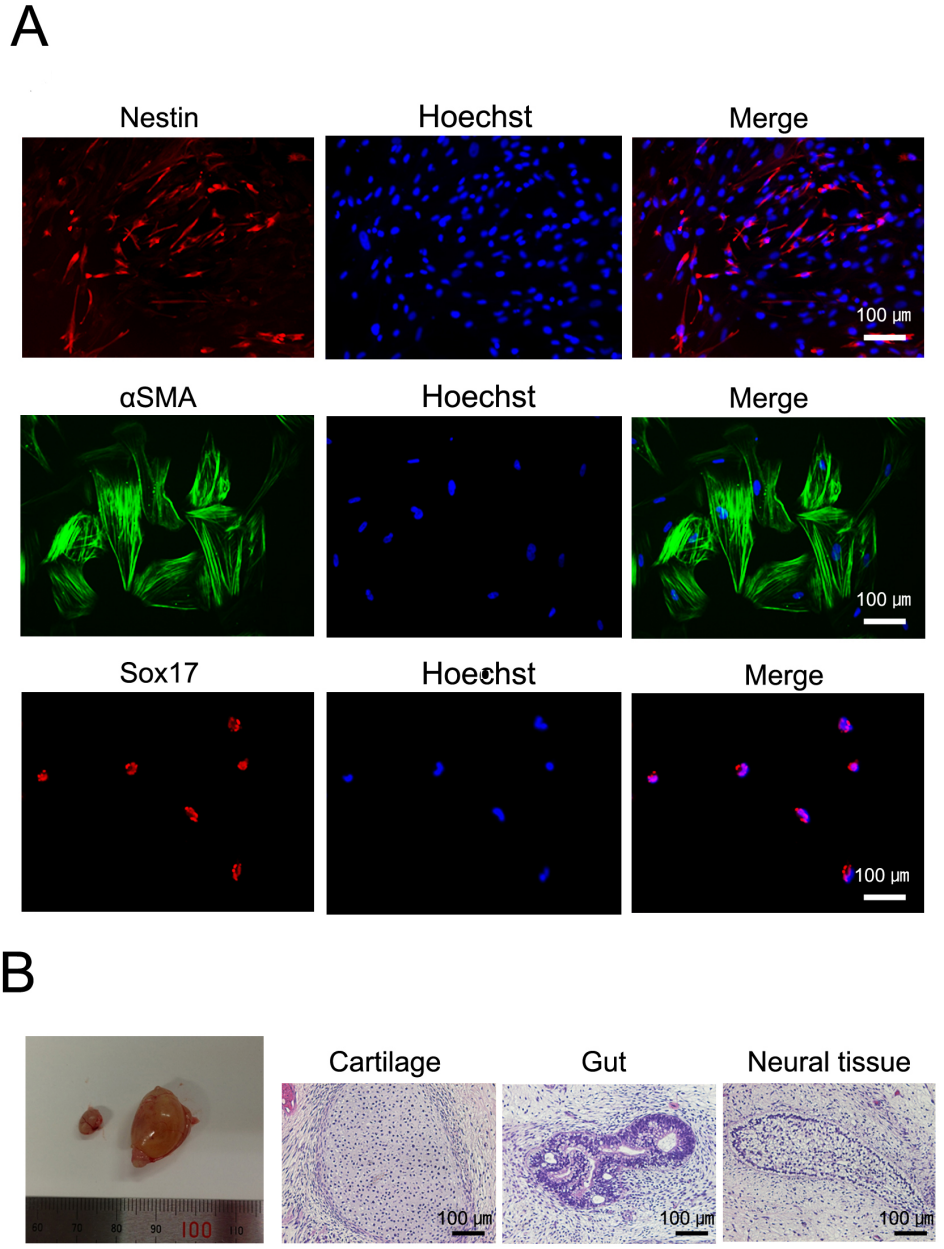
In vitro and in-TiPSCs. (A): Immunofluorescence staining for Sox17 (endodermal marker), αSMA (mesodermal marker), and Nestin (ectodermal marker) in each TiPSCs1-derived differentiated cell in vitro. (B): Gross morphology of representative teratomas derived from TiPSCs1 in vivo (hematoxylin and eosin staining).

To then demonstrate the pluripotency of our M-TiPSCs, we checked their differentiation capability. In vitro differentiation assays revealed M-TiPSC-generated embryoid bodies that contained derivatives of all three germ layers (Fig. 4A, Fig.S1B). In addition, M-TiPSCs injected into the testes of SCID mice gave rise to teratomas that contained derivatives of all three germ layers (Fig. 4B). These results indicate that the M-TiPSCs generated herein are pluripotent stem cells.

## Discussion

In this report, we successfully generated iPSCs from human peripheral T cells without fetal calf serum in the culture medium and MEF as a feeder layer. These substrates are associated with the possibility of the transfer of exogenous antigens, unknown viruses, or zoonotic pathogens to the cell populations generated[15]. Although our protocol uses some substrates (e.g. Matrigel[30], anti-CD3 mAb, CTK solution[31], and SeV[32]) that are associated with a risk of transferring animal-derived pathogens, reducing the number of animal-derived substrates in the culture system will have a significant impact on the clinical application of iPSCs. However, the use of such products must be managed with strict quality control to avoid the risk of pathogen transfer and/or, in the future, these products should be removed from the protocol.

T cells are clinically suitable for iPSC generation because of their ease of sampling and culturing[5]. T cells have been successfully reprogrammed into a pluripotent state with retrovirus[7], [10], lentivirus[4], [6], or Sendai virus[5] using MEF feeder layers for the reprogramming culture conditions. Here, we report using a combination of SeV and activated T cells to successfully reprogram human T cells into a pluripotent state using defined culture conditions with verification of rearranged TCR genes. For clinical applications of iPSCs, establishment of such conditions are highly desirable for maximal safety and feasibility. Although reactivation of exogenous genes in the iPSCs still carries a risk of tumorigenesis[33], RNA-based vectors such as Sendai virus are much safer in this respect than DNA-based vectors, because they never integrate into a host genome[11]–[13]. The resultant feeder-independent TiPSCs thus have greatly enhanced safety for use in regenerative medicine.

Moreover TiPSCs have two unique features that are advantageous for clinical applications. First, because each TiPSC line contains a single rearranged TCR gene in its genome[4]–[7], they can be applied to novel immune therapy. Indeed, successful differentiation of human TiPSCs generated from antigen-specific T cells was reported[8], [9]. Second, the rearrangement patterns of T-cell receptor loci are readily traceable to identify the clonality and the descendants of TiPSCs without insertion of exogenous marker genes[5]. Therefore, generating human TiPSCs under a defined culture condition in which the potential risk of unpredictable agents is reduced, is valuable to extend applications of TiPSCs and is significant for implementing regenerative therapies using iPSCs.

In conclusion, we developed a method for safer iPSCs generation using Sendai virus and activated T cells under a defined culture condition for clinical use. This reprogramming system also provides advantages for the development of novel immune therapy and genetic markers for use as a clinical research tool for future applications of regenerative medicine.

## Supporting Information

Figure S1 **Characterization of M-TiPSCs2 generated under defined culture conditions.** (A): ALP and immunofluorescence staining for pluripotency and surface markers (NANOG, OCT3/4, SSEA3, SSEA4, TRA-1–60, and TRA-1–81) in M-TiPSCs2. (B): Immunofluorescence staining for Sox17 (endodermal marker), αSMA (mesodermal marker), and Nestin (ectodermal marker) in each TiPSCs2-derived differentiated cell in vitro.
(TIF)

Table S1 **Oligonucleotide primers used for PCR.**
(DOCX)
