# Supplementary figures and images for: Derivation of Transgene-Free Human Induced Pluripotent Stem Cells from Human Peripheral T Cells in Defined Culture Conditions
Source: PLoS One. 2014 May 13;9(5):e97397. doi: 10.1371/journal.pone.0097397 (PMC4019536; doi:10.1371/journal.pone.0097397)

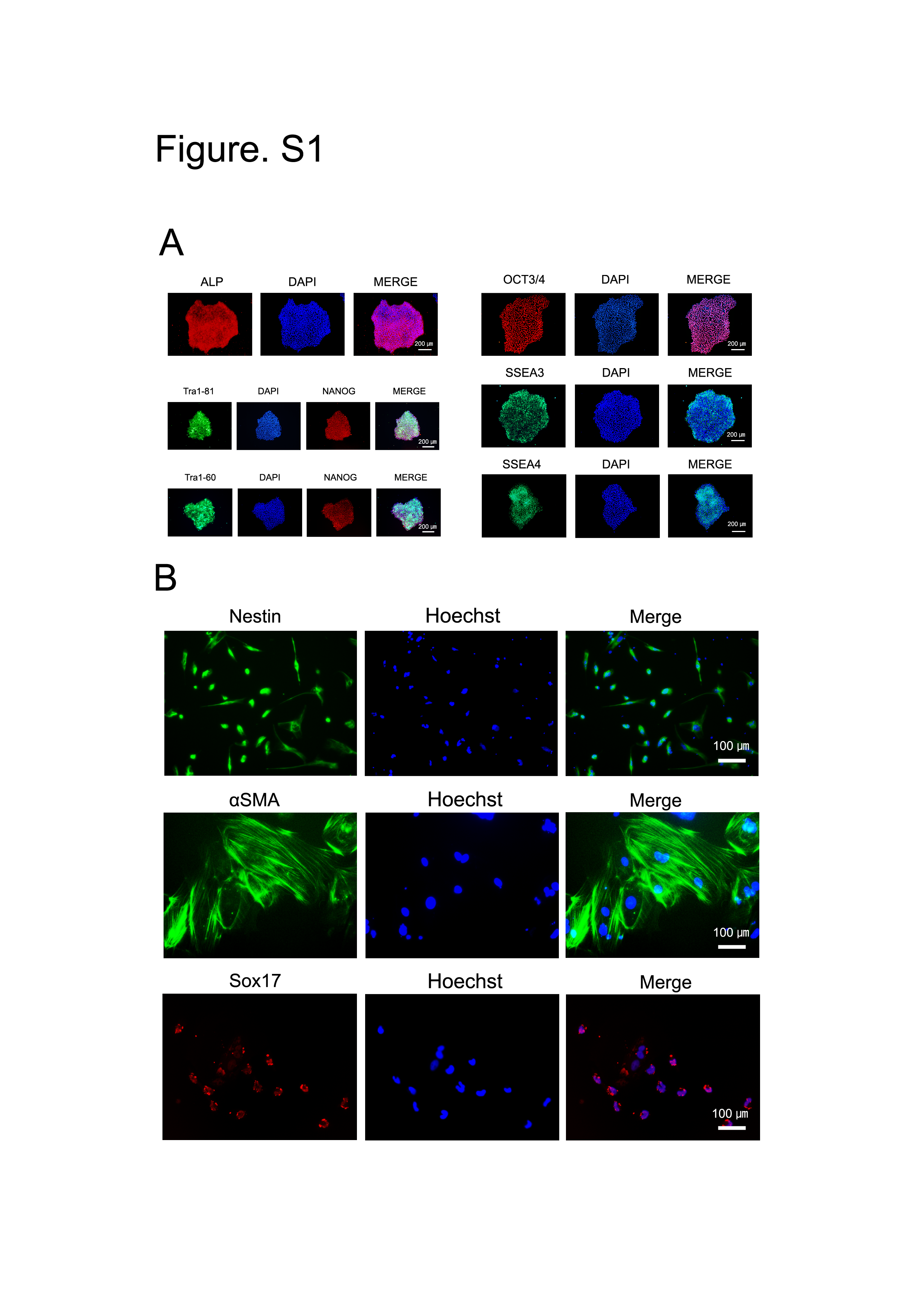

Supplement: Figure S1 — Characterization of M-TiPSCs2 generated under defined culture conditions. (A): ALP and immunofluorescence staining for pluripotency and surface markers (NANOG, OCT3/4, SSEA3, SSEA4, TRA-1–60, and TRA-1–81) in M-TiPSCs2. (B): Immunofluorescence staining for Sox17 (endodermal marker), αSMA (mesodermal marker), and Nestin (ectodermal marker) in each TiPSCs2-derived differentiated cell in vitro. (TIF) [file pone.0097397.s001.tif]
