# Supplementary material for: Derivation of Transgene-Free Human Induced Pluripotent Stem Cells from Human Peripheral T Cells in Defined Culture Conditions
Source: PLoS One. 2014 May 13;9(5):e97397. doi: 10.1371/journal.pone.0097397 (PMC4019536; doi:10.1371/journal.pone.0097397)
Supplement: Table S1 — Oligonucleotide primers used for PCR. (DOCX) [file pone.0097397.s002.docx]

**Table S1: Oligonucleotide primers used for PCR**

| **Target genes** | **Primer sequence (forward)** | **Primer sequence (reverse)** | **AT (°C)** | **Number of cycles** |
| --- | --- | --- | --- | --- |
| *hOCT3/4* (endogenous) | GACAGGGGGAGGGGAGGAGCTAGG | CTTCCCTCCAACCAGTTGCCCCAAAC | 56 | 35 |
| *hSOX2* (endogenous) | GGGAAATGGGAGGGGTGCAAAAGAGG | TTGCGTGAGTGTGGATGGGATTGGTG | 56 | 35 |
| *hKLF4* (endogenous) | ACGATCGTGGCCCCGGAAAAGGACC | TGATTGTAGTGCTTTCTGGCTGGGCTCC | 56 | 35 |
| *hMYC* (endogenous) | GCGTCCTGGGAAGGGAGATCCGGAGC | TTGAGGGGCATCGTCGCGGGAGGCTG | 56 | 35 |
| *hNANOG* | CAGCCCCGATTCTTCCACCAGTCCC | CGGAAGATTCCCAGTCGGGTTCACC | 56 | 35 |
| *hOCT3/4* (SeV transgene) | CCCGAAAGAGAAAGCGAACCAG | AATGTATCGAAGGTGCTCAA | 54 | 35 |
| *hSOX2* (SeV transgene) | ACAAGAGAAAAAACATGTATGG | ATGCGCTGGTTCACGCCCGCGCCCAGG | 54 | 35 |
| *hKLF4* (SeV transgene) | ACAAGAGAAAAAACATGTATGG | CGCGCTGGCAGGGCCGCTGCTCGAC | 54 | 35 |
| *hMYC* (SeV HNL transgene) | TAACTGACTAGCAGGCTTGTCG | TCCACATACAGTCCTGGATGATGATG | 54 | 35 |
| *GAPDH* | CAGAACATCATCCCTGCCTCTAG | TTGAAGTCAGAGGAGACCACCTG | 60 | 35 |
| *SP6* promoter primer | ATTTAGGTGACACTATAGAA |  | 50 | 25 |
| *hOCT3*/4 (converted) | GAGGTTGGAGTAGAAGGATTGTTTTGGTTT | CCCCCCTAACCCATCACCTCCACCACCTAA | 60 | 43 |
| *hOCT3*/4 (unconverted) | GAGGCTGGAGCAGAAGGATTGCTTTGGCCC | CCCCCCTGGCCCATCACCTCCACCACCTGG | 60 | 43 |
| *hNANOG* (converted) | TGGTTAGGTTGGTTTTAAATTTTTG | AACCCACCCTTATAAATTCTCAATTA | 60 | 43 |
| *hNANOG* (unconverted) | TGGCCAGGCTGGTTTCAAACTCCTG | GACCCACCCTTGTGAATTCTCAGTTA | 60 | 43 |

AT, annealing temperature.
